# PUMA–p53 Dysregulation and Ki-67 Overexpression Define Unfavorable Prognostic Signatures in Colorectal Cancer

**DOI:** 10.3390/cancers18010072

**Published:** 2025-12-25

**Authors:** Alexandros Mekras, Dimitrios Tsavdaris, Dimosthenis Mekras, Alexandra Vasilakou, Daniel Paramythiotis, Antonios Michalopoulos, Mattheos Bobos

**Affiliations:** 1 Department of General and Visceral Surgery, SHG-Klinikum Merzig, Academic Hospital of University of Saarland, 66663 Merzig, Germany; 2 First Propaedeutic Surgery Department, University General Hospital of Thessaloniki AHEPA, Aristotle University of Thessaloniki, 54636 Thessaloniki, Greecedanosprx@auth.gr (D.P.); amichal@auth.gr (A.M.); 3 Department of Biomedical Sciences, School of Medical Sciences, International Hellenic University, Sindos, 57400 Thessaloniki, Greece; mbobos@icloud.com

**Keywords:** anti-apoptotic, cancer specific survival, colorectal cancer, mdm2, overall survival, right colon cancer, prognostic indicators, p53, PUMA

## Abstract

**Background/Objectives**: Colorectal cancer (CRC) is the third most commonly occurring cancer. Apoptosis is a fundamental cellular process of programmed cell death, and although many pathways for inducing apoptosis may exist, only the intrinsic and the extrinsic pathways have been demonstrated in detail. This study investigated the expression of BAD, BID, BCL2, MDM2, p53, Ki-67, and PUMA in primary CRC, paired lymph node metastases, and adjacent normal mucosa and explored their associations with clinicopathologic features and patient outcomes. **Methods**: One hundred thirty patients who underwent surgery for resectable CRC were included in the study. FFPE tumor tissue samples were prospectively collected and used for the construction of the TMA blocks from the primary tumor, paired lymph node metastasis, and paired normal mucosa. Immunohistochemistry for BAD, BID, BCL2, MDM2, p53, Ki-67, and PUMA antibodies was performed. **Results**: Univariate analysis showed reduced cancer-specific (CSS), disease-free (DFS), and overall survival (OS) in patients with lymphatic invasion, ≥4 positive lymph nodes, poorly differentiated tumors, older age (≥65), right-sided tumors, stage IIIC disease, or no chemotherapy. Multivariate analysis identified lymphovascular invasion, ≥4 metastatic nodes, and high Ki-67 as independent predictors of worse DFS and CSS, with low BAD expression additionally predicting DFS. For OS, adverse predictors included nodal burden, tumor location, absence of chemotherapy, and high p53, MDM2, and Ki-67 expression. Notably, combined high PUMA and low p53 expression independently predicted poorer CSS and OS. **Conclusions**: High expression of PUMA combined with low expression of p53 and a high expression of Ki-67 were independent unfavorable prognostic indicators for both OS and CSS. Further studies are required to clarify the prognostic and therapeutic role of these markers in CRC.

## Simple Summary

This study evaluated key apoptotic regulators (BAD, BID, BCL2, MDM2, p53, PUMA) and the proliferation marker Ki-67 in stage II–III colorectal cancer to determine their prognostic relevance. We found that high Ki-67 expression and a combined signature of high PUMA with low p53 independently predicted inferior overall and cancer-specific survival. Furthermore, low BAD expression was associated with an increased risk of recurrence. These results emphasize a specific apoptosis–proliferation dysregulation profile that may further improve prognostic stratification beyond conventional clinicopathologic factors and promote biomarker-guided management strategies in colorectal cancer.

## 1. Introduction

Colorectal cancer (CRC) is among the most common malignancies worldwide [1]. While surgical resection is the primary treatment for patients with stages I–III CRC, adjuvant treatment with 5-fluorouracil (5-FU)-based chemotherapy with the addition of oxaliplatin (FOLFOX/XELOX regimen) in stage III disease is estimated to result in an approximately 5–6% improvement in 5-year overall survival (OS) [2].

The process of tumor development in CRC is thoroughly studied and well-characterized, and although early detection, improved treatment regimens, and increased understanding of the disease have contributed to declining mortality rates over the past decade, the survival of CRC patients still largely depends on disease stage at diagnosis and varies widely between stages [3,4]. In clinical practice, while TNM staging remains the cornerstone for clinical decision-making, additional molecular and pathological factors (e.g., microsatellite instability, KRAS/BRAF mutation status) are increasingly integrated into practice and research [5].

Apoptosis, a fundamental process of programmed cell death, maintains tissue homeostasis and prevents the survival of damaged cells. Evasion of apoptosis is a hallmark of CRC progression and treatment resistance [6]. Two major pathways regulate apoptosis: the intrinsic (mitochondrial) and extrinsic (death receptor) pathways [7]. The intrinsic pathway involves mitochondrial cytochrome c release and caspase activation, tightly controlled by the Bcl-2 protein family, which includes both anti-apoptotic (BCL-2, BCL-XL, MCL-1, etc.) and pro-apoptotic (BAX, BAK, BID, BIM, BAD) members that share conserved BH domains [8]. BH3-only proteins (Bad, Bid, Bim, Puma, and Noxa) are sensors of cellular damage and can engage and inactivate prosurvival Bcl-2 family members [9]. The interaction of BH3-only proteins through their BH3 domain with prosurvival proteins negates their cytoprotective function.

The TP53 tumor suppressor plays a central role in DNA damage response and apoptosis regulation [10]. The TP53 tumor suppressor induces apoptosis by upregulating the expression of the Noxa and Puma BH3-only proteins, doing so in response to substantial levels of DNA breaks and other chromosomal abnormalities [10]. Furthermore, MDM2 oncoprotein (mouse double minute 2, HDM2 in humans) binds directly to the N-terminus transactivation domain of p53, regulates the transcriptional activity of p53, and indicates the nuclear export of p53 [11]. MDM2 regulates the ubiquitination of p53, which leads to its degradation, and this forms a negative feedback loop that maintains low levels of p53 in normal cells [12]. Recent research in CRC further indicates that oncogenic drivers such as KIF20A and the long non-coding RNA LINC01836 can disrupt apoptosis and tumor-suppressive signaling, in part by altering metabolic and transcriptional pathways that intersect with p53-mediated cell cycle control [13,14].

Ki-67 is a nuclear protein associated with cell proliferation [15,16]. It is associated with transcription of ribosomal RNA 210 and its inactivation leads to inhibition of synthesis [16]. The Ki-67 marker is present in all proliferating cells and expressed in all phases of the cell cycle, except the resting phase (G0 phase), and there is a great interest in its role as an proliferations marker [17]. The percentage of Ki-67 positive cells in a tumor is called Ki-67 labeling index (LI) and is often associated with the clinical course of the disease. In colorectal cancer (CRC), the prognostic significance of Ki-67 expression remains less consistent. While numerous studies and meta-analyses report that elevated Ki-67 expression is associated with reduced overall and disease-free survival, others have observed a paradoxical association with improved outcomes in specific subgroups [18,19,20].

While Bcl-2 and p53 molecules have been widely investigated in CRC by different researchers, contradictory observations concerning their expression and prognostic value have been reported [21,22,23]. To date, only sparse data are available about the expression and prognostic value of other members of pro-apoptotic pathway, such as Bad, Bid, Puma, and the p53 pathway or MDM2 in CRC.

The aim of this study was to investigate the expression of BAD, BID, BCL2, MDM2, p53, Ki67, and PUMA in primary CRC and paired locally metastatic and normal colon epithelium. Furthermore, we investigated the potential associations of all of the above-mentioned molecules with each other, when appropriate, and with clinical and pathologic characteristics and outcomes.

## 2. Materials and Methods

### 2.1. Patients

One hundred thirty patients who underwent surgery for resectable CRC were included in the study. Eligibility criteria for this study were as follows: a: histologically confirmed CRC with anatomic and clinical TNM, UICC/AJCC stage II and III (8th edition, 2016); b: adequacy of clinical data on patient history, demographics, tumor characteristics, treatment details, and clinical outcome; and c: availability of adequate formalin-fixed paraffin-embedded (FFPE) tumor tissue for biological marker evaluation.

The research protocol was approved by the Bioethics Committee of the School of Medicine, Aristotle University, Thessaloniki (protocol #232; 23 March 2016).

### 2.2. Tissue Processing and Tissue Microarray (TMA) Construction

**Figure 1:**
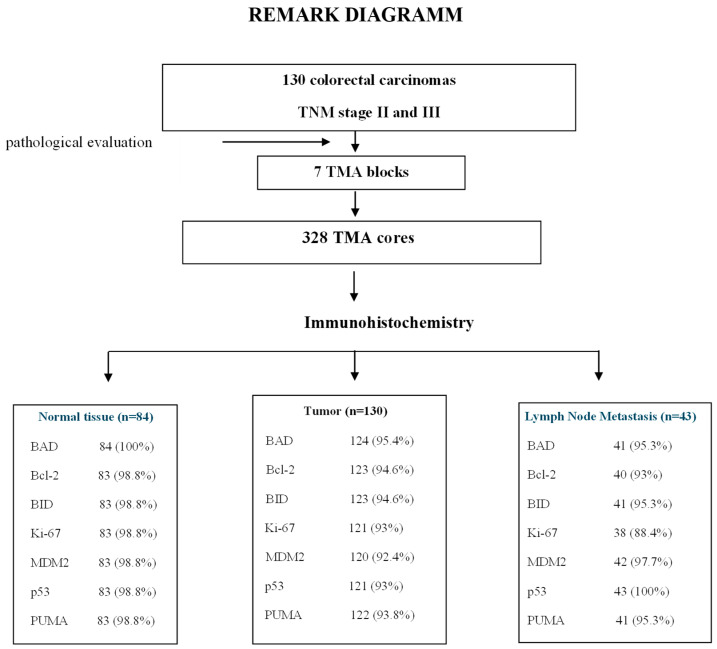
Flowchart depicting the inclusion and tissue microarray (TMA) construction process for this colorectal cancer (CRC) study. A total of 130 patients with histologically confirmed stage II or III CRC were included. Formalin-fixed paraffin-embedded tissue samples from primary tumors, paired lymph node metastases (*n* = 43), and adjacent normal mucosa (*n* = 84) were collected and assembled into 7 TMA blocks containing 328 tissue cores. Immunohistochemical analysis was performed for apoptotic and proliferation markers BAD, BCL2, BID, Ki-67, MDM2, p53, and PUMA. Numbers and percentages reflect the successful evaluation rate of each marker in the respective tissue types. This diagram illustrates the comprehensive coverage of samples and markers used for biomarker expression and correlation analyses in the study.

Formalin-fixed paraffin-embedded (FFPE) tumor tissue samples were prospectively collected and histologically reviewed for tissue and tumor availability by an experienced pathologist (MB) and the most representative areas were marked for the construction of the TMA blocks with the use of a manual arrayer (Model I, Beecher Instruments, San Prairie, WI, USA). In total, 328 FFPE tissue blocks from 130 patients were collected. These samples included the primary tumor, paired lymph node metastases (LNM) in 43 cases and paired normal mucosa (N) in 84 cases. Two 1.5 mm cores per tumor, as well as one core from cases with paired normal mucosa and one core from the paired LNMs, were transferred into 7 low density tissue microarrays (TMAs), which were used for in situ methods. The TMA blocks contained a total of 594 tissue cores. In each TMA block, 10 tissue cores from neoplastic and non-neoplastic tissues were also included as positive and negative controls of the tested antibodies. To assure optimal immunoreactivity, immunostaining was performed 7–10 days after sectioning at the Laboratory of Molecular Oncology of the Hellenic Foundation for Cancer Research, School of Medicine, Aristotle University of Thessaloniki. The REMARK diagram for the study is shown in Figure 1.

### 2.3. Immunohistochemistry (IHC) and Interpretation of IHC Results

Serial 3 μm thick TMA sections were stained for BAD, BID, BCL2, MDM2, p53, PUMA, and Ki67 antibodies on Bond-Max autostainer. Diaminobenzidine (DAB) was used as a chromogen and Mayer’s hematoxylin as a counterstain. The sources of the antibodies and the conditions of staining were as shown in Table 1. All sections were stained in one run for each antibody and were evaluated by one of the authors (MB) blinded as to the patient’s clinical characteristics and survival data.

For each immunostain, cellular localization of the protein, i.e., the nucleus or cytoplasm, was recorded. The expression of BAD, BID, BCL2, MDM2, p53, and PUMA was calculated based on the intensity of staining (I) on a scale of 0–3 (0 = no expression, 1 = mild expression, 2 = moderate expression, 3 = intense expression), whereas percentage of positive neoplastic cells (P) for each localization was also scored on a scale of 0–100%. The histoscore (H-Score) was then created according to the formula 1 × P + 2 × P + 3 × P = 0–300. The H-Score in each case was calculated from the average of the H-Score in the two tissue cylinders, i.e., TC and IF. If one of the tissue cores was lost or damaged, the score was determined from the remaining core. The Ki-67 marker was evaluated as a percentage of positive cells from the “hot spots” of the tumor, in a range from 0–100.

### 2.4. Statistical Analysis

The median H-Score was used as a predetermined cut-off for each antibody. Positive samples or samples with high expression were considered when the value of H-Score was equal to or greater than the median. We used the median H-Score as the default cut-off value for each indicator, and this was performed to avoid false-positive results from multiple calculations of cut-offs. After specifying the median value for each sample, additional and combinatorial variables were created. χ^2^ or Fisher’s exact tests and Mann–Whitney or Kruskall–Wallis tests were used to test associations between protein expression and different categorical clinicopathological variables. OS was defined as the time from the date of diagnosis to the date of death regardless of cause and DFS as the time elapsed between the date of diagnosis and the date of the patient’s confirmed recurrence of illness [24]. Disease-specific survival (DSS) or CSS was stated as the time elapsed from the date of diagnosis to the date of death from CRC [24]. Survival probabilities were estimated by the Kaplan–Meier method and compared using the log-rank test. Model choice was performed using backward selection criteria at *p* < 0.15.

Analyses were conducted using the SAS software (version 9.3, SAS Institute Inc., Cary, NC, USA) and the language R, specific to statistical computations and graphics (version 2.14.1, “R & R”, Statistics Department of the University of Auckland). The results of this study are presented according to reporting recommendations for tumor marker prognostic studies [18].

## 3. Results

### 3.1. Clinicopathologic Features and Tumor Characteristics

The mean patient age was 69.8 years (SD, 10.5) in a range of 32–91 years. The majority of patients were age ≥ 65 years (93, 71.6%) (*p* = 0.0001) and were male (71, 54.6%). Of all patients, 39 (30%) had cancer in the right colon (cecum, ascending colon, right colic flexure, and proximal transverse colon 2/3), 52 (40%) in the left colon (left colic flexure, descending colon, and sigmoid), and 39 (30%) in the rectum. Large tumors >3 cm were found in 91 (70%) of patients, mainly in the rectum, in 30 out or 39 patients (77%). The majority of tumors showed moderate histologic differentiation (71.6%). Of the patients, 50% were classified according to Astler–Coller classification as Stage B2, while the remaining 50% as Stage C. Lymphovascular invasion was detected in 30% of cases, while 50% of cases were free of nodal metastases (*p* = 0.009). A total of 21.6% of cases had >4 metastatic lymph nodes (LNs). Sixty-five patients (50%) were classified as AJCC stage III; the majority had rectum rather than colon cancer, present in twenty-four and forty-one patients, respectively (*p* = 0.058).

In 88 (67.7%) patients, postoperative chemotherapy was administered; 40 (30.8%) received no treatment other than surgical excision of the tumor, while for 2 (1.5%), postoperative information was not available. Fifty-five patients received FOLFOX, ten received patients 5FU-LV, and twenty-three patients received XELOX as a postoperative chemotherapy treatment.

Clinicopathological features of the studied patients and their resected colorectal carcinomas are shown in Table 2.

### 3.2. Protein Marker Expression by Immunohistochemistry (IHC)

The proportions of eligible cases tested by IHC for protein evaluation expression were >92% for tumors, >98.5% for normal tissues, and >90.5% for metastatic tumors (REMARK diagram, Figure 1).

**Figure 2:**
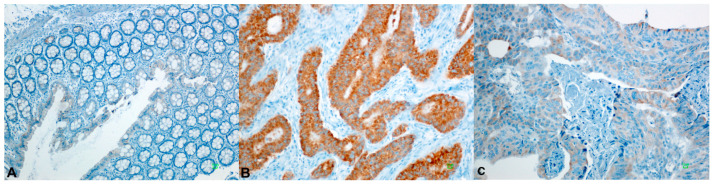
Underexpression (**A**) and high expression (**B**) of MDM2 protein in normal and tumor tissue, respectively. (**C**) Diffuse low expression of MDM2.

**Figure 3:**
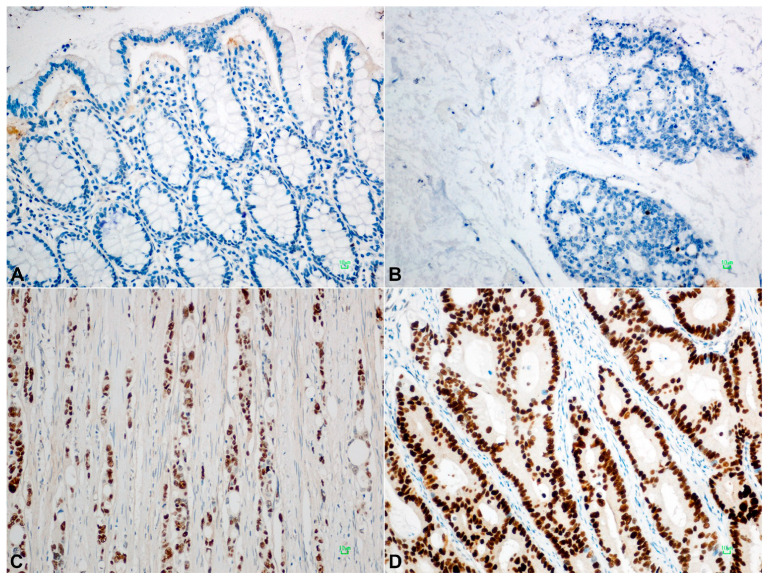
(**A**) Lack of P53 expression in normal colonic mucosa. (**B**) Null pattern of expression. (**C**) Low expression/wild type. (**D**) Strong nuclear staining: >90% cells denotes overexpression, signifying an underlying TP53 mutation.

**Figure 4:**
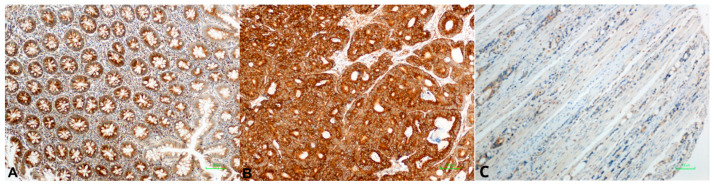
PUMA expression in normal colonic mucosa and tumor tissue. (**A**) Low expression of PUMA protein in normal mucosa and PUMA overexpression in tumor (**B**). (**C**) Mild to moderate expression of PUMA in <75% of tumor cells.

High BAD expression was detected in 53/124 (43%) primary tumors, in 18/41 (44%) LN metastases, and in 40/84 (48%) normal colonic mucosal tissues. BAD staining showed a lower median H-Score on normal tissues than primary neoplastic or locally metastatic tumors, i.e., a 100 median H-Score for normal colonic mucosa, 140 for LN tumor metastasis (*p* = NS), 170 for TC (*p* = 0.003), and 190 for IF (*p* = 0.001). BID overexpression was identified in 55/123 (45%) of primary CRC tumors, in 6/41 (15%) LN metastases, and in 26/83 (41%) normal mucosa cases. The median BID expression was two-fold higher in tumors and metastases than normal tissue, with a median H-Score of 100 for normal tissue, 200 for LN metastasis (*p* = 0.001), 200 for TC (*p* = 0.001), and 200 for IF (*p* = 0.001) (Elevated Bcl2 protein levels were observed in 60/123 (49%) tested primary tumors, in 20/40 (50%) LN metastases, and in almost half of normal mucosa cases (40/83, 48%). A higher median H-Score for BCL2 was noticed in normal tissues compared to primary and metastatic tumors, while high values of BCL2 expression, over 200, were detected only in tumor tissues. BCL2 expression in LN, IF, and TC was statistically significant compared to normal tissue (*p* = 0.003, *p* = 0.001 and *p* = 0.001, respectively). MDM2 protein was expressed in 60/120 (50%) CRC cases, 21/42 (50%) LN metastases, and 39/83 (47%) of cases with a normal epithelium. Higher median H-Scores of MDM2 were detected in tumors compared to normal tissue and metastatic tumors, whereas the highest values of MDM2 expression (H-Score: 300) were detected only in tumor tissues (Figure 2 and Figure S1). Tumor protein p53 showed overexpression in 58/121 (48%) tested CRCs. A similar number of LN metastatic cases showed elevated p53 expression (21/43, 49%). A high H-Score of p53 staining in normal tissues (>220) was noticed in only three cases (3.6%). P53 staining showed lower median H-Scores in normal tissues than primary neoplastic or locally metastatic tumors, i.e., a median H-Score of 5 for normal colonic mucosa, 160 for LN metastasis (*p* = 0.001), 150 for TC (*p* = 0.001), and 170 for IF (*p* = 0.001) (Figure 3). We noticed high PUMA protein expression in 82/122 (67%) primary CRC cases, in 20/41 (49%) LN metastases, and in 27/83 (32.5%) normal tissues. Higher median H-Scores of PUMA were detected in primary and metastatic tumors compared to normal tissue, with statistical significance only in TC (*p* = 0.001) and IF (*p* = 0.012) (Figure 4 and Figure S1).

Finally, Ki67 proliferation was high in 60/120 (50%) CRCs, 17/38 (45%) LN metastases, and 30/83 (36%) of non-neoplastic mucosa cases.

Representative immunohistochemical staining patterns demonstrating high expression of BAD, BID, BCL2, MDM2, p53, Ki-67, and PUMA in colorectal carcinoma are shown in Figure 2 and Figure 3.

### 3.3. Correlations Between Molecules and Clinicopathological Variables

Statistically significant correlations emerged between the expression of BAD/BID (*p* = 0.001), PUMA/BAD (*p* = 0.018), PUMA/Ki-67 (*p* <0.0001), and p53/MDM2 (*p* = 0.033) in primary tumors, as detailed in Table 3.

Significant associations were noticed between BCL2 and tumor differentiation (*p* = 0.036), BAD and tumor localization (*p* = 0.011), p53 and the number of infiltrated lymph nodes (*p* = 0.024), BID and maximal tumor diameter and lymphovascular invasion (*p* = 0.002 and *p* = 0.049, respectively), MDM2 and maximal tumor diameter (*p* = 0.015), and Ki-67 and mucinous adenocarcinomas (*p* = 0.016). None of the studied immunohistochemical markers showed a statistically significant correlation with chemotherapy. Full results are presented in Table 4.

Regarding protein expression only on the tumor front (IF), statistically significant correlations were found between LN metastases and BAD and BID molecules (*p* = 0.032 and *p* = 0.032, respectively), histological differentiation and BAD (*p* = 0.052), maximum tumor diameter and BID and Ki-67 (*p* = 0.030 and *p* = 0.033, respectively), Astler–Coller stage and p53 (*p* = 0.020), as well as between the number of filtered lymph nodes (and stage N by TNM) and the expression of p53 protein (*p* = 0.001) and between the end of mucosal adenocarcinomas and the MDM2 molecule (*p* = 0.009).

### 3.4. Survival and Prognostic Factors

During a median follow-up of 54 months, a total of 26 recurrence events were recorded. Of the overall deaths, 16 were attributed to non-colorectal cancer causes. The 3-year DFS was 67%, while for OS and CSS, the progression of the disease was identical (70.8%).

Univariate survival analysis showed significantly smaller CSS, DFS, and OS for patients with lymphatic invasion (*p* = 0.0031, *p* = 0.0072 and *p* = 0.0289) and ≥4 infiltrated lymph nodes (*p* = 0.0016, *p* = 0.0051 and *p* < 0.0001). Moreover, patients with poorly differentiated tumors had worse CSS (*p* = 0.0074). Significantly lower OS was observed in patients who did not receive chemotherapy (*p* < 0.0001), in patients aged ≥ 65 years (*p* = 0.0109), in AJCC stage IIIC patients (*p* = 0.0035), and in those with adenocarcinoma in the right colon (*p* = 0.0309).

**Figure 5:**
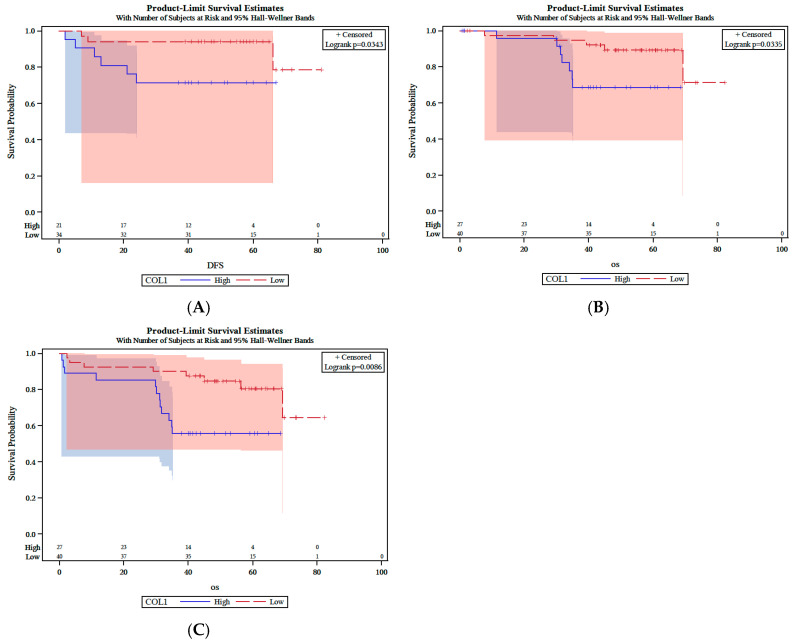
Kaplan–Meier survival analysis. Low BAD expression in primary tumors was associated with lower DFS (**A**), inferior CSS (**B**), and poorer OS (**C**).

Low expression of BAD in IF of primary tumors was associated with worse DFS (*p* = 0.034), lower CSS (*p* = 0.0086), and lower OS in patients (*p* = 0.033) (Figure 5A–C). Low Ki-67 expression was significantly associated with longer OS (*p* = 0.03) Similarly, the combination of low expression of p53 and PUMA in TC of primary tumors showed a marginally significant association with OS (*p* = 0.068). Finally, patients whose tumors showed low expression of PUMA were associated with a marginally shorter OS (*p* = 0.056).

On multivariate analysis, lymphovascular invasion (HR = 4.17 and HR = 2.72), ≥4 infiltrated lymph nodes (HR = 1.39 and HR = 1.79), and high Ki-67 labeling index (HR = 3.20 and HR = 2.36) were highlighted as independent unfavorable prognostic indicators for both DFS and CSS. In addition, low expression of BAD was an independent adverse predictor for DFS (HR = 2.97). For OS, ≥4 metastatic lymph nodes (HR = 4.89), the location of the primary tumor in the rectum and right colon (HR = 5.05, HR = 2.63), no chemotherapy treatment (HR = 7.36), and high-expression of p53, MDM2, and Ki-67 (HR = 0.35, HR = 2, and HR = 2.94) emerged as an independent adverse prognostic indicators. Finally, the combination of high expression of PUMA with low expression of p53 was highlighted as having an independent unfavorable prognostic value on both CSS and OS (HR: 2.42, CI 0.6–9.7, Wald *p* = 0.0503 and HR: 4.6, CI 0.85–24.98, Wald *p* = 0.0963, respectively) (Table 5 and Table 6).

## 4. Discussion

This study investigated the immunohistochemical expression patterns of key apoptotic regulators (BAD, BID, BCL2, MDM2, p53, and PUMA) and the proliferation marker Ki-67 in resectable stage II–III colorectal cancer (CRC) and evaluated their potential clinicopathological and prognostic significance. The findings provide evidence that deregulation of apoptosis- and proliferation-related proteins plays a critical role in colorectal carcinogenesis and disease progression, while specific profiles may serve as independent prognostic markers.

In our work, the location of the primary tumor had a significant effect on prognosis. Univariate analysis showed a lower OS and a strong positive trend of poorer CSS for patients with CRC and tumor localization in the right colon (RC). Multivariate analysis demonstrated that tumor localization in the RC and rectum is an independent adverse prognostic indicator. On the other hand, tumor localization in the left colon (LC) was identified as a favorable prognostic indicator.

Univariate analysis also showed significantly lower CSS, DFS, and OS in patients with angiolymphatic infiltration (*p* = 0.0031) or neoplastic infiltration of equal or more than four LNs (N2 per TNM). The same clinicopathological factors also emerged as independent adverse prognostic indicators in the multivariate analysis for DFS and CSS. Our study showed that each of the above two factors (LN metastasis and lymphovascular invasion) has an independent prognostic value for 3-year survival (CSS, DFS, and OS) in patients with stage II and III colon cancer.

Furthermore, in our study, there was a statistically significantly lower CSS in patients with low tumor differentiation, while for DFS, there was a strong positive trend of poor prognosis for the same characteristic. In our study’s multivariate analysis of DFS, CSS, and OS, high levels of Ki-67 expression emerged as an adverse prognostic indicator for stage II and III patients. Ki-67 was overexpressed in lymph node metastases compared to normal tissue.

We also observed increased BAD expression in primary tumors compared to normal tissue, both at the tumor center and invasive margin, while a difference was found, but without being statistically significant, between its expression in LN metastases and the normal intestinal epithelium. BAD overexpression was an independent adverse prognostic factor for DFS and was significant in univariate analysis for DFS, CSS, and OS. In its active dephosphorylated form, BAD is dimerized with either Bcl-2 or Bcl-XL, preventing their binding to Bax, thus promoting apoptosis. However, no correlation was observed between BAD and Bcl-2 expression, though the two molecules showed nearly identical distribution at the invasive margin, indicating disrupted apoptosis in CRC.

BAD protein showed a strong positive correlation with PUMA, suggesting apoptosis induction, as PUMA acts as a promoter of both p53-dependent and p53-independent apoptosis.

Significant differences were observed in BAD, PUMA, and p53 expression between normal mucosa and carcinomas (tumor center and invasive margin), but not in lymph node metastases. DNA damage from gamma radiation or chemotherapeutics (e.g., 5-FU, adriamycin, etoposide) induces p53-dependent PUMA expression and apoptosis. The combination of PUMA overexpression and p53 underexpression was identified as an independent adverse predictor for OS and CSS, likely reflecting a disruption of the balance between proliferation and apoptosis in CRC. Finally, we observed that MDM2 overexpression was associated with adverse OS in CRC.

Our results on apoptosis regulators and proliferation markers in general concur with earlier reports in stage II–III colorectal cancer. Overexpression of p53 and high Ki-67 were associated with poor prognosis, consistent with a number of studies demonstrating their independent prognostic value for relapse-free and overall survival [22,25]. Overexpression of BCL2 was associated with favorable outcomes, consistent with reports that BCL2 positivity is predictive of early-stage, well-differentiated tumors [26,27]. BAD and PUMA expression was upregulated in tumor tissue compared to normal mucosa, with significant positive correlation between them, reflecting their pro-apoptotic function; however, similar to earlier studies, BAD expression was not correlated with BCL2, suggesting autonomous regulation of apoptosis in CRC [28,29,30]. Surprisingly, overexpression of BAD was found to be an independent negative prognostic factor for DFS, suggesting that dysregulation of pro-apoptotic signaling may contribute to aggressive tumor behavior [30].

Regarding clinicopathological variables, our findings confirm the adverse prognostic significance of lymphovascular invasion, N2 node status, and adverse tumor differentiation for CSS, DFS, and OS, in agreement with large series reports and database analyses [31,32,33,34,35,36,37,38]. Improved outcomes for right-sided colon cancers were consistent with previous findings in stage III–IV disease, although the impact in stage II is less clear-cut [39,40,41,42]. Both high Ki-67 values and low differentiation were also associated with poor tumor biology [22,25]. MDM2 overexpression was also associated with poor OS, consistent with its role in p53 degradation and chemoresistance [43,44]. In contrast, PUMA overexpression with low p53 as a poor predictor is not in line with most studies in the literature, which generally portray low PUMA expression as an omen for poor outcomes [45,46,47]. Combined, our findings substantiate the prognostic role of apoptotic regulators, proliferation markers, and conventional clinicopathological features in stage II–III CRC, suggesting the potential utility of incorporating these biomarkers into clinical decision-making and risk stratification. Rectal tumors in our cohort showed a higher prevalence of nodal metastasis and greater exposure to adjuvant chemotherapy (Table 2), which may introduce residual confounding when colon and rectal cases are analyzed together; although tumor location was included among the variables tested in multivariable models (and was an independent predictor for overall survival), formal stratified or interaction analyses by anatomic site were not performed and should be considered in future validation studies to exclude site-driven effects.

The findings of this study have significant clinical implications for stage II–III colorectal cancer prognostication and potential therapeutic decision-making. Identification of combined high PUMA and low p53 expression, as well as a high Ki-67 proliferative index, as unfavorable independent prognostic factors predicts that these molecular markers might add value to conventional clinicopathologic risk stratification. Inclusion of these biomarkers in clinical algorithms might have the potential to enable more precise selection of patients at a higher risk of recurrence or poor survival who would be eligible for intensified surveillance or consideration of adjuvant therapy. Also, the observed correlation between impaired apoptosis, namely by the PUMA–p53 pathway, and aggressive tumor behavior justifies developing treatments against such pathways. If validated, modalities of treatment that focus on modulation of the p53–PUMA regulatory pathway or control of proliferative function could represent novel precision medicine in the treatment of colorectal cancer. It should be emphasized that the term ‘low p53 expression’ used in this study refers solely to the immunohistochemical phenotype. Reduced or weak p53 staining does not necessarily correspond to the TP53-null molecular subtype, as low protein expression may occur in tumors with wild-type TP53 or with mutations that do not eliminate protein production. The unfavorable prognosis observed in cases with high PUMA expression and low p53 expression should therefore be interpreted as a functional, phenotype-level pattern of impaired apoptotic signaling, rather than as a direct reflection of TP53-null status.

The paradoxical association of high PUMA expression with low p53 expression as an adverse prognostic pattern can be understood better in light of recent evidence on p53-independent apoptotic regulation. While PUMA is classically induced by functional p53, it has also been reported to be upregulated via alternative stress-responsive pathways [22,32,48,49]. In the event of defective downstream apoptotic machinery or enhanced anti-apoptotic signaling, however, high levels of PUMA expression do not directly correlate with effective cell death. By contrast, low p53 often reflects TP53 deletion, loss-of-function mutation, or enhanced degradation, all of which are associated with aggressive tumor behavior in CRC and perhaps override any pro-apoptotic influence of PUMA [22,34,49,50,51] Cohort-specific patterns may include BAX dysfunction, altered BCL-2 family activity, or post-translational disruption of PUMA, any of which may further uncouple PUMA abundance from apoptotic competence [22,48,49]. Mechanistically, these considerations parallel established evidence showing that a high Ki-67 proliferation index is predictive of recurrence and poor survival, with some reports of differential response to adjuvant therapy. Moreover, a growing number of studies indicates that the use of apoptotic markers such as p53, PUMA, and BCL-2 family proteins in combination can enhance risk stratification beyond conventional staging, although further validation is required before its routine clinical use [22,48,49,50,51,52]. Taken together, these findings provide a contextual framework for our results and support the concept of integrating apoptotic and proliferative markers in colorectal cancer prognostication.

This study benefits from a well-characterized cohort with clearly defined inclusion and exclusion criteria, focusing specifically on resectable stage II–III colorectal cancer and including comprehensive clinical and pathological annotation. The use of a robust tissue microarray platform allowed high-throughput, standardized immunohistochemical assessment across paired tumor, lymph node metastasis, and adjacent normal tissue samples, while blinded evaluation minimized observer bias. Prognostic correlations were rigorously analyzed by univariate and multivariate models, and findings were put into perspective with the literature to include enhanced interpretive information. This study has several limitations. It was conducted at a single institution with a moderate-sized cohort, which may limit generalizability, and the findings require validation in larger, multi-center studies. The cross-sectional analysis of protein expression precludes inference of causality or mechanistic pathways. Immunohistochemical evaluation was performed by a single experienced pathologist; while most markers showed broadly consistent staining within tumor cores, some proteins exhibited modest intra-tumoral variation. TMA sampling may not capture absolute intratumoral heterogeneity, and Ki-67 scoring did not use standardized hotspot-oriented field selection, limiting assessment of proliferative gradients. Additionally, the longitudinal orientation of normal colonic crypts was not controlled, so evaluation of apoptotic and proliferative markers in non-tumoral mucosa should be interpreted with caution. Certain biomarker combinations, such as high PUMA–low p53, differed from previous reports, possibly due to cohort-specific effects or methodological differences. Limited mutational and molecular data (e.g., TP53 sequence, MSI status, KRAS/BRAF mutations) restricted inclusion of genetic context and molecular subtypes. H-scores were used to capture combined staining intensity and distribution but may be less practical for widespread clinical use; simplified thresholds based on the proportion of cells with at least moderate staining could improve feasibility and reproducibility. Predictive utility for responsiveness to specific therapies was not assessed, so findings should be interpreted as strictly prognostic rather than predictive. Finally, multiple statistical comparisons were conducted within a single training cohort without independent validation; associations should therefore be regarded as exploratory and hypothesis-generating, requiring confirmation in larger external cohorts with appropriate correction for multiple testing.

## 5. Conclusions

In this study, we demonstrated that dysregulation of apoptotic signaling, particularly through the PUMA–p53 axis, and increased proliferative activity, marked by Ki-67 overexpression, are independently associated with adverse outcomes in colorectal cancer. High PUMA expression combined with low p53 expression, along with elevated Ki-67, correlated with significantly poorer overall and cancer-specific survival, underscoring the critical interplay between apoptosis resistance and tumor proliferation in disease progression. Low BAD expression further identified patients at increased risk of recurrence, emphasizing its potential role as a tumor-suppressive marker. Collectively, these findings highlight a distinct molecular signature involving PUMA, p53, BAD, and Ki-67 that may refine prognostic stratification beyond conventional clinicopathologic factors. Future studies integrating molecular and functional analyses are warranted to validate these markers as potential prognostic tools and therapeutic targets in colorectal cancer.

## Figures and Tables

**Table 1:** Antibodies and staining procedures used in the present study.

Antigen	Clone/P	AR/Τ/WB	AD	I	DTS/P	SP
BAD	ab32445, Rabbit polyclonal (1)	ER1/HP, 20′/PBS	1:250	30′	Polymer HRP/DAB (3)	C
Bcl2	124, Mouse monoclonal (2)	ER1/HP, 20′/PBS	1:80	20′	Polymer HRP/DAB (3)	C
BID	ab32060, Rabbit polyclonal (1)	ER1/HP, 20′/PBS	1:100	15	Polymer HRP/DAB (3)	C
Ki-67	MIB1, Mouse monoclonal (2)	ER2/HP, 20′/PBS	1:70	70′	Polymer HRP/DAB (3)	N
MDM2	SMP14, Mouse monoclonal (1)	ER2/HP, 20′/PBS	1:50	30′	Envision (2)	C/N
p53	DO-7, Mouse monoclonal (2)	ER2/HP, 20′/PBS	1:100	30′	Polymer HRP/DAB (3)	N
PUMA	ab 33906, Rabbit polyclonal (1)	ER1/HP, 20′/PBS	1:30	30′	Polymer HRP/DAB (3)	C

Abbreviations: AD: antibody dilution; AR: antigen retrieval; C: cytoplasm; DTS: detection system; ER1: citric acid pH6.0; ER2: Ethylenediaminetetraacetate, pH8.8; N: nuclear; P: provider; PBS: phosphate-buffered saline. Antibody and detection system providers: (1): Abcam, Cambridge, UK; (2): Dako, Glostup, Denmark; (3): Leica Biosystems, Newcastle Upon Tyne, UK.

**Table 2:** Patient and tumor characteristics in the entire cohort and in the colon and rectum cancer groups.

		Total	Colon	Rectum	*p*-Value
N		130 (100%)	91 (70%)	39 (30%)	
Age					
	Mean (SD)	69.8 (10.5)	72.4 (9.7)	63.7 (9.7)	
	Min–Max	32–91	49–91	32–79	
Age					
	<65	37 (28.4%)	18 (19.8%)	19 (48.8%)	0.001
	≥65	93 (71.6%)	73 (80.2%)	20 (51.2%)	
Sex					
	Female	59 (45.4%)	43 (47.2%)	16 (41%)	0.513
	Male	71 (54.6%)	48 (52.8%)	23 (59%)	
Tumor primary site					
	Rectum	39 (30%)		39 (100%)	-
	Left colon	52 (40%)	52 (57.2%)	-	-
	Right colon	39 (30%)	39 (42.8%)	-	-
Tumor size					
	≤3	39 (30%)	30 (33%)	9 (23%)	0.259
	>3	91 (70%)	61 (67%)	30 (77%)	
LVI					
	Yes	39 (30%)	24 (26.4%)	15 (38.4%)	0.168
	No	91 (70%)	67 (73.6%)	24 (61.6%)	
Grade					
	Low	16 (12.4%)	12 (13.2%)	4 (10.2%)	0.363
	High	21 (16.2%)	12 (13.2%)	9 (23%)	
	Intermediate	93 (71.6%)	67 (73.6%)	26 (66.6%)	
Astler–Coller					
Classification	B2	65 (50%)	50 (55%)	15 (38.4%)	0.085
	C1-C2	65 (50%)	41 (45%)	24 (61.6%)	
T(TNM)					
	T2	6 (4.6%)	4 (4.4%)	2 (5.2%)	0.855
	T3-T4	124 (95.4%)	87 (95.6%)	37 (94.8%)	
N(ΤΝΜ)					
	N0	65 (50%)	50 (55%)	15 (38.4%)	0.009
	N1	37 (28.4%)	28 (30.8%)	9 (23%)	
	N2	28 (21.6%)	13 (14.2%)	15 (38.4%)	
AJCC Stage					
	IIA	65 (50%)	50 (55%)	15 (38.4%)	0.058
	IIIA	4 (3%)	3 (3.2%)	1 (2.6%)	
	IIIB	52 (40%)	35 (38.4%)	17 (43.6%)	
	IIIC	9 (7%)	3 (3.2%)	6 (15.4%)	
Recurrence					
	No	99(79.2)	68 (79.1)	31 (79.5)	0.958
	Yes	26(20.8)	18 (20.9)	8 (20.5)	
CTx					
	Yes	88 (67.6%)	54 (59.4%)	34 (87.2%)	0.003
	No	40 (30.8%)	35 (38.4%)	5 (12.8%)	
	Not reported	2 (1.6%)	2 (2.2%)	-	
CTx Schemes					
	Not reported	2 (1.6%)	2 (2.2%)	-	0.026
	FOLFOX	55 (42.4%)	33 (36.2%)	22 (56.4%)	
	None	40 (30.8%)	35 (38.4%)	5 (12.8%)	
	5FU-LV	10 (7.6%)	7 (7.6%)	3 (7.6%)	
	XELOX	23 (17.6%)	14 (15.4%)	9 (23%)	

Abbreviations: CTx: chemotherapy; 5FU-LV: Fluorouracil-Leucovorin Calcium; LVI: lymphovascular invasion.

**Table 3:** Correlations of protein markers in the primary tumor.

		BAD			BCL2			BID			Ki-67			MDM2			p53		
		H	L	*p*	H	L	*p*	H	L	*p*	H	L	*p*	H	L	*p*	H	L	*p*
BCL2	H	30 (56.6)	30 (42.9)	0.131															
	L	23 (43.4)	40 (57.1)																
BID	H	33 (62.3)	22 (31.4)	0.001	26 (43.3)	29 (46.8)													
	L	20 (37.7)	48 (68.6)		34 (56.7)	33 (53.2)													
Ki-67	H	27 (52.9)	25 (35.7)	0.059	28 (47.5)	24 (39.3)	0.37	27 (50.9)	25 (37.3)	0.135									
	L	24 (47.1)	45 (64.3)		31 (52.5)	37 (60.7)		26 (49.1)	42 (62.7)										
MDM2	H	30 (56.6)	30 (44.8)	0.198	32 (53.3)	27 (45.8)	0.409	28 (51.9)	32 (48.5)	0.714	25 (48.1)	33 (50.8)	0.772						
	L	23 (43.4)	37 (55.2)		28 (46.7)	32 (54.2)		26 (48.1)	34 (51.5)		27 (51.9)	32 (49.2)							
p53	H	24 (46.2)	34 (49.3)	0.734	24 (40.7)	33 (54.1)	0.141	28 (51.9)	29 (43.9)	0.388	28 (53.8)	29 (42.6)	0.223	34 (57.6)	22 (37.9)	0.033			
	L	28 (53.8)	35 (50.7)		35 (59.3)	28 (45.9)		26 (48.1)	37 (56.1)		24 (46.2)	39 (57.4)		25 (42.4)	36 (62.1)				
PUMA	H	41 (78.8)	41 (58.6)	0.018	44 (74.6)	38 (61.3)	0.118	40 (74.1)	42 (62.7)	0.183	47 (90.4)	35 (51.5)	0	38 (65.5)	42 (70.0)	0.602	42 (73.7)	40 (64.5)	0.28
	L	11 (21.2)	29 (41.4)		15 (25.4)	24 (38.7)		14 (25.9)	25 (37.3)		5 (9.6)	33 (48.5)		20 (34.5)	18 (30.0)		15 (26.3)	22 (35.5)	

L: low expression; H: high expression.

**Table 4:** Correlations of clinicopathological variables and protein markers expression in the primary tumor.

		MDM2			BID			Ki-67			BAD			BCL2			p53			PUMA		
		High	Low	*p*	High	Low	*p*	High	Low	*p*	High	Low	*p*	High	Low	*p*	High	Low	*p*	High	Low	*p*
CTx	No	20 (37)	18 (27)	0.231	18 (35)	19 (28.4)	0.465	15 (25.0)	22 (37.3)	0.148	15 (28.3)	23 (33.3)	0.552	20 (33.9)	18 (29.0)	0.564	17 (29.3)	20 (32.8)	0.682	28 (34.1)	10 (26.3)	0.391
	Yes	34 (63)	49 (73)		34 (65)	48 (71.6)		45 (75.0)	37 (62.7)		38 (71.7)	46 (66.7)		39 (66.1)	44 (71.0)		41 (70.7)	41 (67.2)		54 (65.9)	28 (73.7)	
CTS	FOLFOX	24 (44)	28 (42)	0.325	24 (46)	28 (41.8)	0.292	26 (43.3)	25 (42.4)	0.379	23 (43.4)	30 (43.5)	0.889	23 (39.0)	30 (48.4)	0.683	26 (44.8)	26 (42.6)	0.247	38 (46.3)	14 (36.8)	0.205
	No	20 (37)	18 (27)		18 (35)	19 (28.4)		15 (25.0)	22 (37.3)		15 (28.3)	23 (33.3)		20 (33.9)	18 (29.0)		17 (29.3)	20 (32.8)		28 (34.1)	10 (26.3)	
	5FU/LV	2 (4)	7 (10)		1 (2)	7 (10.4)		5 (8.3)	4 (6.8)		4 (7.5)	5 (7.2)		4 (6.8)	5 (8.1)		7 (12.1)	2 (3.3)		5 (6.1)	3 (7.9)	
	XELOX	8 (15)	14 (21)		9 (17)	13 (19.4)		14 (23.3)	8 (13.6)		11 (20.8)	11 (15.9)		12 (20.3)	9 (14.5)		8 (13.8)	13 (21.3)		11 (13.4)	11 (28.9)	
Age	<65	12 (22)	22 (32)	0.194	12 (23)	21 (30.4)	0.368	15 (25.0)	18 (30.0)	0.540	17 (32.1)	18 (25.4)	0.411	16 (26.7)	19 (30.2)	0.668	19 (32.8)	15 (23.8)	0.274	20 (24.4)	14 (35.0)	0.220
	≥65	43 (78)	46 (68)		40 (77)	48 (69.6)		45 (75.0)	42 (70.0)		36 (67.9)	53 (74.6)		44 (73.3)	44 (69.8)		39 (67.2)	48 (76.2)		62 (75.6)	26 (65.0)	
G	L	7 (13)	8 (12)	0.568	9 (18)	6 (8.7)	0.340	8 (13.3)	7 (11.7)	0.962	9 (17.0)	6 (8.5)	0.051	6 (10.0)	9 (14.3)	0.036	10 (17.2)	5 (7.9)	0.190	10 (12.2)	5 (12.5)	0.743
	H	11 (20.0)	9 (13)		7 (13)	12 (17.4)		10 (16.7)	10 (16.7)		12 (22.6)	8 (11.3)		5 (8.3)	15 (23.8)		10 (17.2)	8 (12.7)		12 (14.6)	8 (20.0)	
	I	37 (67)	51 (75)		36 (69)	51 (73.9)		42 (70.0)	43 (71.7)		32 (60.4)	57 (80.3)		49 (81.7)	39 (61.9)		38 (65.5)	50 (79.4)		60 (73.2)	27 (67.5)	
LI	No	33 (60)	52 (76.5)	0.049	36 (69)	48 (69.6)	0.968	41 (68.3)	41 (68.3)	1.000	32 (60.4)	54 (76.1)	0.061	45 (75.0)	40 (63.5)	0.167	39 (67.2)	46 (73.0)	0.488	55 (67.1)	29 (72.5)	0.543
	Yes	22 (40)	16 (23.5)		16 (31)	21 (30.4)		19 (31.7)	19 (31.7)		21 (39.6)	17 (23.9)		15 (25.0)	23 (36.5)		19 (32.8)	17 (27.0)		27 (32.9)	11 (27.5)	
MTD (cm)	≤3	25 (45.5)	13 (19)	0.002	22 (42)	15 (21.7)	0.015	16 (26.7)	22 (36.7)	0.239	19 (35.8)	19 (26.8)	0.277	20 (33.3)	18 (28.6)	0.568	19 (32.8)	18 (28.6)	0.617	30 (36.6)	8 (20.0)	0.063
	>3	30 (54.5)	55 (81)		30 (58)	54 (78.3)		44 (73.3)	38 (63.3)		34 (64.2)	52 (73.2)		40 (66.7)	45 (71.4)		39 (67.2)	45 (71.4)		52 (63.4)	32 (80.0)	
Sex	F	25 (45.5)	30 (44)	0.882	26 (50.0)	28 (40.6)	0.302	27 (45.0)	25 (41.7)	0.713	22 (41.5)	33 (46.5)	0.582	28 (46.7)	27 (42.9)	0.671	26 (44.8)	28 (44.4)	0.966	38 (46.3)	16 (40.0)	0.508
	M	30 (54.5)	38 (56)		26 (50.0)	41 (59.4)		33 (55.0)	35 (58.3)		31 (58.5)	38 (53.5)		32 (53.3)	36 (57.1)		32 (55.2)	35 (55.6)		44 (53.7)	24 (60.0)	
AC Cl.	B2	24 (44)	36 (53)	0.305	24 (46)	35 (50.7)	0.619	29 (48.3)	31 (51.7)	0.715	29 (54.7)	32 (45.1)	0.288	33 (55.0)	27 (42.9)	0.178	33 (56.9)	27 (42.9)	0.123	40 (48.8)	20 (50.0)	0.899
	C1-C2	31 (56)	32 (47)		28 (54)	34 (49.3)		31 (51.7)	29 (48.3)		24 (45.3)	39 (54.9)		27 (45.0)	36 (57.1)		25 (43.1)	36 (57.1)		42 (51.2)	20 (50.0)	
ΤΝΜ (N)	N0	24 (44)	36 (53)	0.311	24 (46)	35 (50.7)	0.810	29 (48.3)	31 (51.7)	0.901	29 (54.7)	32 (45.1)	0.132	33 (55.0)	27 (42.9)	0.127	33 (56.9)	27 (42.9)	0.024	40 (48.8)	20 (50.0)	0.992
	N1	15 (27)	20 (29)		15 (29)	20 (29.0)		16 (26.7)	16 (26.7)		10 (18.9)	25 (35.2)		18 (30.0)	17 (27.0)		10 (17.2)	25 (39.7)		23 (28.0)	11 (27.5)	
	N2	16 (29)	12 (18)		13 (25)	14 (20.3)		15 (25.0)	13 (21.7)		14 (26.4)	14 (19.7)		9 (15.0)	19 (30.2)		15 (25.9)	11 (17.5)		19 (23.2)	9 (22.5)	
TMN (T)	T2	2 (4)	4 (6)	0.565	2 (4)	4 (5.8)	0.625	3 (5.0)	3 (5.0)	1.000	2 (3.8)	4 (5.6)	0.633	5 (8.3)	1 (1.6)	0.083	3 (5.2)	3 (4.8)	0.917	3 (3.7)	2 (5.0)	0.726
	T3-T4	53 (96)	64 (94)		50 (96)	65 (94.2)		57 (95.0)	57 (95.0)		51 (96.2)	67 (94.4)		55 (91.7)	62 (98.4)		55 (94.8)	60 (95.2)		79 (96.3)	38 (95.0)	
AJCC	IIA	24 (44)	36 (53)	0.336	24 (46)	35 (50.7)	0.501	29 (48.3)	31 (51.7)	0.753	29 (54.7)	32 (45.1)	0.454	33 (55.0)	27 (42.9)	0.057	33 (56.9)	27 (42.9)	0.412	40 (48.8)	20 (50.0)	0.919
	IIIA	1 (2)	3 (4)		2 (4)	2 (2.9)		3 (5.0)	1 (1.7)		1 (1.9)	3 (4.2)		4 (6.7)			2 (3.4)	2 (3.2)		2 (2.4)	1 (2.5)	
	IIIB	27 (49)	23 (34)		20 (38.5)	29 (42.0)		24 (40.0)	23 (38.3)		18 (34.0)	32 (45.1)		20 (33.3)	30 (47.6)		19 (32.8)	30 (47.6)		33 (40.2)	17 (42.5)	
	IIIC	3 (5)	6 (9)		6 (11.5)	3 (4.3)		4 (6.7)	5 (8.3)		5 (9.4)	4 (5.6)		3 (5.0)	6 (9.5)		4 (6.9)	4 (6.3)		7 (8.5)	2 (5.0)	
TL	Colon	42 (76.4)	45 (66.2)	0.217	39 (75.0)	46 (66.7)	0.321	44 (73.3)	40 (66.7)	0.426	34 (64.2)	53 (74.6)	0.206	43 (71.7)	43 (68.3)	0.680	44 (75.9)	41 (65.1)	0.195	59 (72.0)	26 (65.0)	0.433
	Rectum	13 (23.6)	23 (33.8)		13 (25.0)	23 (33.3)		16 (26.7)	20 (33.3)		19 (35.8)	18 (25.4)		17 (28.3)	20 (31.7)		14 (24.1)	22 (34.9)		23 (28.0)	14 (35.0)	

AC Cl.: Astler–Coller classification; CTx: chemotherapy; L: low grade; H: high grade; I: intermediate grade; LI: lymphovascular invasion; MTD: maximal tumor diameter; F: female; M: male.

**Table 5:** Multivariate Cox regression analysis of CSS including the combined PUMA/p53 variable.

Description	HazardRatio	WaldLower	WaldUpper	ProbChiSq
Stage_N_N1 vs. N0	0.145	0.017	1.277	0.0819
Stage_N_N2(a + b) vs. N0	1.394	0.431	4.510	0.5795
Invasion Yes vs. No	4.166	1.205	14.402	0.0242
KI67_High vs. Low	3.199	1.081	9.461	0.0356
BAD_High vs. Low	2.968	0.934	9.425	0.0651

**Table 6:** Multivariate Cox regression analysis of OS including the combined PUMA/p53 variable.

Description	HazardRatio	WaldLower	WaldUpper	ProbChiSq
Stage_N_N1	0.451	0.113	1.804	0.2601
Stage_N_N2(a + b)	2.843	0.897	9.013	0.0759
Invasion Yes	0.920	0.280	3.018	0.0532
Rectum vs Colon	3.407	1.104	10.510	0.8902
Right vs. Left Colon	2.866	0.985	8.333	0.0330
KI67_High	1.315	0.230	7.505	0.1104
PUMA_p53_high/high	4.599	0.847	24.982	0.7579
PUMA_p53_high/low	2.674	0.382	18.729	0.0772
PUMA_p53_low/high	2.222	0.834	5.924	0.3221

## Data Availability

The data presented in this study contain sensitive patient information and are not publicly available due to ethical and legal restrictions. De-identified data may be obtained from the corresponding author upon reasonable request and following approval by the Bioethics Committee of the School of Medicine, Aristotle University of Thessaloniki.

## Supplementary Materials

The following supporting information can be downloaded at: https://www.mdpi.com/article/10.3390/cancers18010072/s1, Figure S1: Boxplots 1 & 2: PUMA and MDM2 protein expression, relative to the sample point of the tissue.
