# Supplementary material for: PUMA–p53 Dysregulation and Ki-67 Overexpression Define Unfavorable Prognostic Signatures in Colorectal Cancer
Source: Cancers (Basel). 2025 Dec 25;18(1):72. doi: 10.3390/cancers18010072 (PMC12784786; doi:10.3390/cancers18010072)
Supplement: Supplementary file 1 [file cancers-18-00072-s001.zip › cancers-3963894-supplementary.pdf]

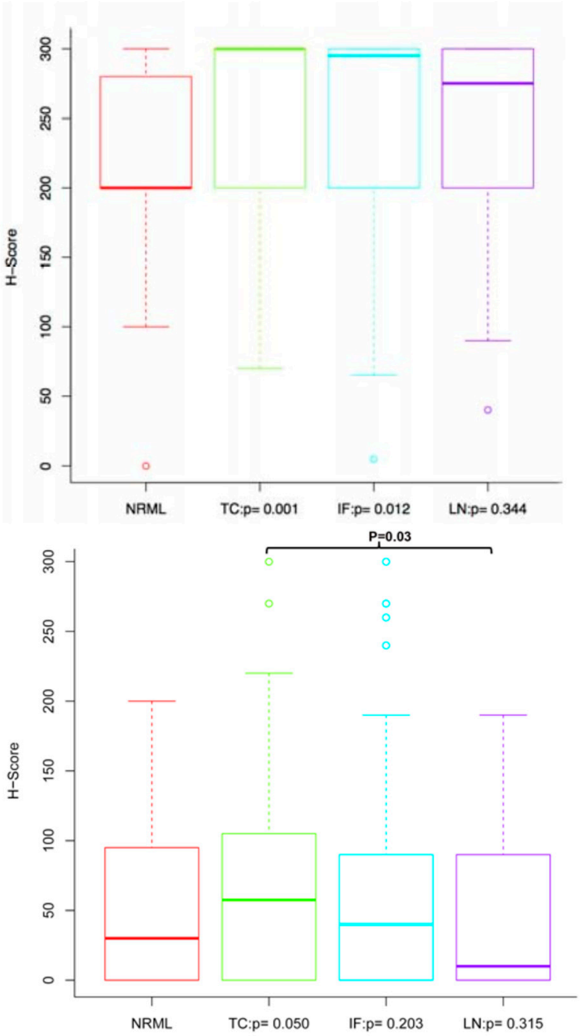

Figure S1. Boxplots 1 & 2: PUMA and MDM2 protein expression, relative to the sample point of the tissue. NRML: Normal tissue; TC: Tumor Core; IF: Invasion front; LN: Lymph node.
